# Impact of a Cryptococcal meningitis diagnosis and treatment program at Lira Regional Referral Hospital in rural, Northern Uganda

**DOI:** 10.1371/journal.pgph.0000254

**Published:** 2022-05-16

**Authors:** Abigail Link, Mark Okwir, David Meya, Betty Nabongo, James Okello, Danuta Kasprzyk, Paul R. Bohjanen

**Affiliations:** 1 University of Washington, School of Nursing, Seattle, WA, United States of America; 2 Division of Infectious Diseases and International Medicine, Department of Medicine, University of Minnesota Medical School, Minneapolis, MN, United States of America; 3 Lira Regional Referral Hospital, Lira, Uganda; 4 Lira University Faculty of Health Sciences, Lira, Uganda; 5 Makerere University, Infectious Diseases Institute, Kampala, Uganda; 6 University of Minnesota School of Public Health, Minneapolis, MN, United States of America; National Institute of Neuroscience Department of Mental Retardation and Birth Defect Research: Kokuritsu Kenkyu Kaihatsu Hojin Seishin Shinkei Iryo Kenkyu Center Shinkei Kenkyujo Shippei Kenkyu Dainibu, BANGLADESH

## Abstract

In rural areas of sub-Saharan Africa, infrastructure and resources for treatment of cryptococcal meningitis (CM) are often lacking. We introduced a CM diagnosis and treatment program (CM-DTP) at Lira Regional Referral Hospital (LRRH) in rural Uganda to determine if implementing high-quality standard of care protocols would improve outcomes. Information extracted from hospital charts and clinical record forms at LRRH were used to compare diagnoses, treatments, and outcomes for all patients diagnosed with meningitis (n = 281) over a two-year period after initiation of the CM-DTP in February of 2017 to all patients diagnosed with meningitis (n = 215) in the two preceding years. After implementation of the CM-DTP, we observed increased confirmed diagnoses of CM from 22.2% (48 of 215) to 35.2% (99 of 281), (p = 0.002) among all patients diagnosed with meningitis. Among all patients treated for CM, the proportion who received standard of care treatment with amphotericin B plus fluconazole increased from 63 of 127 (49.6%) to 109 of 146 (74.7%), (p <0.001) and mortality improved from 66 of 127 (52.0%) to 57 of 146 (39.0%), (p = 0.04) after implementation of the CM-DTP. Implementation of the CM-DTP was associated with increased number of lumbar punctures and decreased use of antibiotics in patients with CM, as well as decreased mortality among patients with meningitis from all causes. Improved diagnosis, treatment, and mortality were observed following implementation of the CM-DTP. Our results demonstrate that quality treatment of CM in rural Uganda is feasible.

## Introduction

Cryptococcal meningitis (CM) is a major cause of death among the 38 million people infected with HIV [1]. This type of meningitis accounts for approximately 15% of AIDS-related deaths, with most deaths occurring in sub-Saharan Africa [2,3]. Despite increased availability of HIV treatment and improved CM therapies, CM kills approximately 181,000 persons per year, with a death rate of 50–70% [4–6]. In Uganda, CM is the second most common cause of AIDS-related death [3]. Although effective treatments for CM are now available in urban centers in sub-Saharan Africa [7], adequate training and resources for diagnosis and treatment of CM are not found on much of the continent [8–11], especially in resource-limited rural areas where most people live [12]. The burden of CM in rural areas is still poorly understood, and recent advances in CM diagnosis and treatment have not been fully implemented in much of sub-Saharan Africa.

In Uganda, approximately 76% of the population resides in rural areas of the country [13]. In Kampala, the capital of Uganda, health care infrastructure and resources are at a relatively high level, contrasted to rural areas where care is often suboptimal because of lack of infrastructure and resources. Without appropriate diagnostic testing, CM diagnosis in rural areas is often made solely on clinical judgement, which can lead to misdiagnoses, improper treatment and poor outcomes. Also, availability of antifungal medications, such as amphotericin B deoxycholate (amphotericin B), appropriate fluid and electrolyte replacement, and laboratory monitoring are often lacking. Because of the challenges for diagnosing and effectively treating CM in rural areas, treatment based on standard of care guidelines is difficult to implement.

In 2016, we assessed CM care at Lira Regional Referral Hospital (LRRH) in rural, northern Uganda and found that diagnostic testing for CM was unavailable within the hospital due to insufficient supplies and was only available for patients who could afford testing at private laboratories outside the hospital. Further, many patients who were treated for CM received suboptimal therapy because the drugs used as standard of care were unavailable within the hospital and had to be purchased from private pharmacies. Only a subset of patients could afford diagnostic testing and standard of care treatments. Our assessment suggested that the diagnosis and treatment of CM at LRRH could be improved, and we wanted to determine if effective treatment of CM was feasible.

Our findings prompted the development a CM Diagnosis and Treatment Program (CM-DTP) at LRRH, which was initiated in February, 2017 with care provided by local clinicians. The program provided diagnostic testing for patients with suspected CM and provided antifungal treatment with amphotericin B and fluconazole, as well as intravenous hydration, electrolyte supplementation, and laboratory monitoring for those diagnosed with CM. The goal of the program was to determine if high quality CM care (currently provided in urban areas) could be implemented by local clinicians in this rural setting to improve outcomes. We assessed the two-year impact of the CM-DTP on diagnosis, treatment, and mortality of patients with CM compared to a historical group of patients from the two years immediately before the CM-DTP.

## Methods

### Ethics statement

The required permits and approvals for performing research on human subjects in Uganda were obtained. Ethics approval was obtained from Gulu University Research Ethics Committee (GUREC) [066–19], Uganda National Council of Science and Technology (UNCST) [HS 2675], University of Washington Institutional Review Board (IRB) [STUDY00007770], and University of Minnesota IRB [STUDY00011386]. The requirement for consent was waived and approved by GUREC and UNCST as the data was extracted from existing paper records and hospital files.

### Study setting

This study was performed at LRRH which is located in northern Uganda and serves approximately 2.2 million people within the nine districts surrounding the hospital [14]. This conflict-torn area was occupied by the Lord’s Resistance Army from 1987 to 2006 [15] but has since stabilized, although health care infrastructure and resources remain relatively poor compared to urban areas of Uganda. Health care infrastructure in northern Uganda was negatively impacted by civil conflict, but at the time the CM-DTP was initiated the situation at LRRH regarding CM diagnosis and care was representative of much of rural Uganda, where resources are often lacking.

### Study design

This retrospective group study compared data from patients with meningitis who were treated from February 2015-February 2017 before the initiation of the CM-DTP (group 1) and February 2017-February 2019 after initiation of the CM-DTP (group 2). Standard of care protocols provided by the CM-DTP were based on Ugandan clinical guidelines [16] and included amphotericin B for 7–14 days, fluconazole, IV fluids, therapeutic LPs, electrolyte supplementation, and laboratory monitoring. Patients in both groups received care by local clinicians. However, the initiation of the CM-DTP increased the workforce for CM care by providing salary support for two part-time medical officers and one full-time nurse.

**Fig 1:**
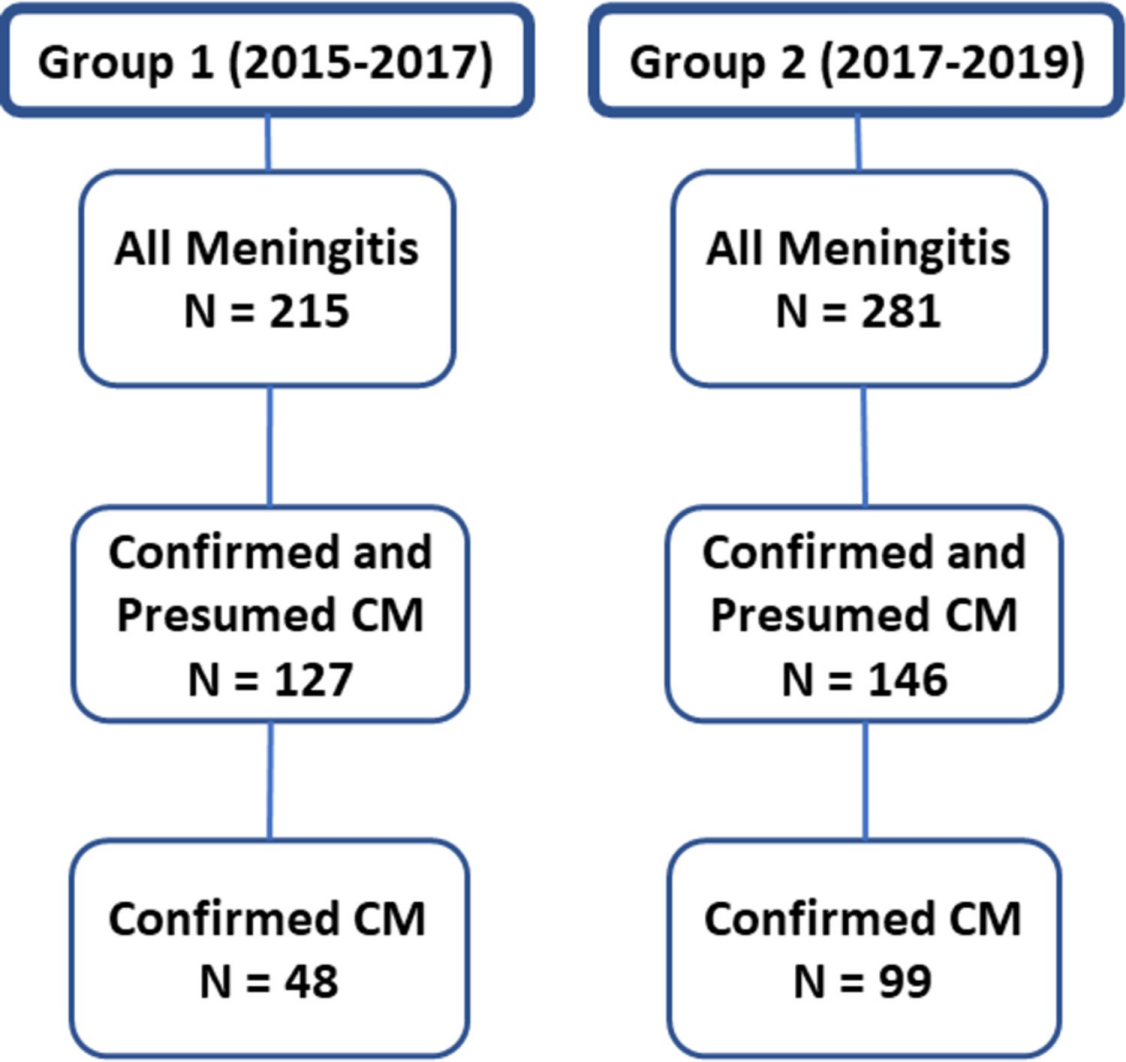


Retrospective chart reviews were conducted on all patients in the adult medical wards at LRRH to identify all patients with any form of meningitis. We reviewed charts of all patients with symptoms of headache in combination with one or more of the following: fever, confusion, photophobia, neck pain/stiffness, seizure, or a Glasgow coma scale (GCS) <15. All patients with CM and meningitis were included over a two-year period for both groups, thus representing the entire population of patients during these periods. From this review, we identified 215 patients in group 1 (hospitalized between January 2015-February 12, 2017) and 281 patients in group 2 (hospitalized between February 13, 2017-February 2019) who had a diagnosis of meningitis (Fig 1), after excluding those who had alternative diagnoses like malaria, hepatic encephalopathy, space occupying lesions, etc. All patients with confirmed CM were ≥14 years old, except one patient in group 2 who was nine-years old.

Data was previously collected on clinical record forms at the time of their care from patients who participated in the CM-DTP related to diagnosis, symptoms, symptom days, treatments, medical history, physical exams, laboratory results, HIV and antiretroviral therapy (ART) status, complications, diagnosis, treatment, and mortality outcomes. For patients in group 1, similar data was collected directly from hospital charts. All information was entered into a database, which was validated and double-checked by a second person.

### Diagnosis of CM

Group 1 patients were considered to have confirmed CM if they had a documented positive cryptococcal antigen (CrAg) test, cerebral spinal fluid (CSF) India ink, or CSF gram stain showing yeast. In group 1, a variety of commercial CrAg latex agglutination (LA) tests were performed at independent laboratories at the patient’s expense. For group 2, the CrAg lateral flow assay (LFA) test (Immy Inc., Norman, Oklahoma), was used exclusively and performed free of charge on blood, serum and/or CSF for CM diagnosis. In some cases, India ink and gram stains were also performed.

For group 1, the CrAg LFA test was not available, and diagnostic testing was often not performed because patients were unable to afford testing. During this time period, the diagnosis of CM was often made based on clinical judgement. Patients were considered to have presumed CM if they had a clinical CM diagnosis (non-laboratory confirmed) recorded in their chart and received antifungal medications for treatment of CM.

### Analyses

We compared groups 1 and 2 among those with laboratory-confirmed CM, those who were treated with antifungal therapy for CM (presumed plus confirmed CM) and those with meningitis of all causes. Descriptive statistics were used to calculate means and proportions on demographic information, admission and discharge diagnoses, baseline neurologic status, symptoms, complications and treatments. To determine significant relationships between the two groups, Chi-square test or Fisher’s Exact test was performed for categorical variables, while Student’s t-test was used for continuous variables. Logistic regression was used to compare mortality with continuous and categorical covariates. Hypothesis testing was two-sided with a significance level of 0.05. All analyses were performed using R version 3.6.0. [17].

## Results

### Patient characteristics

We identified 215 and 281 patients with meningitis in groups 1 and 2, respectively (Fig 1). The primary data used for this manuscript regarding patients with confirmed plus presumed CM are shown in S1 Table, and the primary data regarding patients with non-CM meningitis are shown in S2 Table. Demographic information for all patients with meningitis showed slightly younger patients, fewer males, and fewer patients with previously documented HIV in group 1 versus group 2 (Table 1). In the confirmed plus presumed CM groups, groups 1 and 2 had similar mean age and HIV status, but group 1 had fewer males. Those with HIV had lower mean CD4 counts in group 1 compared to group 2, but similar history of ART experience.

**Table 1:** Demographics of groups 1 & 2.

Group	Group 1 2015–2017	Group 2 2017–2019
All Meningitis	N = 215	N = 281
Age (mean, IQR)	33.6 (26.0–34.2)	35.6 (27.0–44.0)
Gender (Male) (N, %)	93 (43.3**%**)	139 (49.5**%**)
Documented HIV Positive (N, %)	129 (60.0**%**)	234 (83.3**%**)
**Confirmed and Presumed CM**	**N = 127**	**N = 146**
Age (mean, IQR)	36.7 (30.0–45.0)	36.2 (28.0–43.8)
Gender (Male) (N, %)	49 (38.6**%**)	62 (42.5**%**)
Documented HIV Positive (N, %)	127 (100**%**)	145 (99.3%)
CD4 Count (mean, IQR)	69.9 (10.0–96.0)	170.4 (31.5–267.5)
History of ART (N, %)	95 (74.8**%**)	113 (77.4**%**)
Currently on ART (N, %)	92 (72.4**%**)	103 (70.5**%**)
**Confirmed CM**	**N = 48**	**N = 99**
Age (mean, IQR)	36.5 (29.0–45.3)	35.3 (27.0–43.0)
Gender (Male) (N, %)	31 (64.6%)	62 (62.6%)
Documented HIV Positive (N, %)	48 (100%)	99 (100%)
CD4 Count (mean, IQR)	89.8 (23.5–23.5)	129.5 (16.5–161.0)
History of ART (N, %)	37 (77.1%)	79 (79.8%)
Currently on ART (N, %)	37 (77.1%)	70 (70.7%)

Patients were considered to have confirmed CM if they had laboratory diagnosis of CM as described in the methods. The diagnosis of confirmed CM in group 1 was often suboptimal because diagnostic testing was usually only performed for patients who could afford testing at independent laboratories and the available tests had lower sensitivity and specificity compared to the CrAg LFA. For group 2, CM testing by CrAg LFA was provided free of charge to all patients with meningitis, and therefore, more patients were tested. Demographics of patients with confirmed CM were similar between groups with respect to age, gender, and HIV status. At the time of hospitalization, more people in group 1 were currently taking ART, whereas group 2 had more people with a history of ART but some were not currently taking it. Those in group 1 had more episodes of vomiting and confusion compared to group 2 (Table 2). Other clinical characteristics of patients, including fever, headache, GCS, nuchal rigidity, Kernig’s sign, history of CM, history of TB and days of symptoms did not differ significantly between groups.

**Table 2:** Baseline symptoms at admission among CM confirmed.

Characteristics	Group 1 (N = 48) N (%)	Group 2 (N = 99) N (%)	P-value
Headache	41/43 (95.3%)	92/98 (93.9%)	1.00
Fever	24/29 (82.8%)	84/97 (86.6%)	0.56
Vomiting	15/20 (75.0%)	44/92 (47.8%)	**0.05**
Confusion*	18/23 (78.3%)	25/88 (28.4%)	**<0.001**
GCS <15*	10/27 (37.0%)	45/91 (49.5%)	0.28
Nuchal Rigidity	20/30 (66.7%)	77/97 (79.4%)	0.22
Kernig’s Sign	16/23 (69.6%)	64/92 (69.6%)	1.00
CM History	5/48 (10.4%)	11/90 (12.2%)	1.00
TB History	8/46 (17.4%)	24/95 (25.3%)	0.39
Symptom Days	16.4 IQR: 4.3–21.8)	22.3 (IQR: 9.0–28.0)	0.10

*Denominators are not equal in each category due to missing/undocumented data in patient charts. For example, in some charts, confusion only, GCS only, or both confusion and GCS were documented.

### Improved diagnosis of CM after implementation of the CM-DTP

The type of diagnostic tests used to confirm CM for both groups are shown in Table 3. For group 2, 91% of the confirmed CM cases were diagnosed with the CrAg LFA test test (Immy, Norman, Oklahoma) on CSF. The CrAg LFA assay is a rapid and inexpensive diagnostic test for CM that can be performed at the bedside. For group 1, 69% of confirmed CM cases were diagnosed with a CrAg LA test test (Immy, Norman, Oklahoma), while other diagnostic tests were used less frequently.

**Table 3:** CM diagnostic tests among CM confirmed patients.

Diagnostic Tests	Group 1 N (%)	Group 2 N (%)
CSF CrAg Test	33Ψ (68.8%)	90* (90.9%)
Blood CrAg	15Ψ (31.2%)	9* (9.1%)
India Ink Test	13 (27.1%)	51 (51.5%)
Gram Stain Yeast +	20 (41.7%)	47 (47.5%)

*Lateral flow Assay.

Ψ Latex Agglutination.

For patients in group 2, free CrAg LFA testing was available for all patients with meningitis, and most patients in the group who met criteria, received this test. The implementation of this testing led to an increased number of confirmed cases of CM. The number of patients with confirmed CM was 48 out of 215 meningitis patients (22.2%) in group 1 and 99 out of 281 meningitis patients (35.2%) for group 2 (Table 4). Among all cases of meningitis, patients in group 2 had 1.89 times the odds of having a confirmed CM diagnosis compared to those in group 1 (p = 0.002, CI: 1.24–2.90). These finding indicate that implementation of free CrAg LFA testing through the CM-DTP led to increased confirmed diagnoses of CM.

**Table 4:** Impact of the CM-DTP on diagnosis.

Confirmed CM	Group 1 2015–2017		Group 2 2017–2019			
**Diagnosis**	**N**	**%**	**N**	**%**	**P-value**	**OR, CI**
Confirmed CM diagnoses	48	22.3	99	35.2	**0.002**	**1.89, 1.24-2.90**
All cases of meningitis	215		281			

### Improved treatment of CM after implementation of the CM-DTP

Ugandan clinical guidelines for the treatment of CM at the time of this study recommended the use of combination therapy with amphotericin B and fluconazole. Although fluconazole was generally available at LRRH, amphotericin B was not widely available due to hospital stock shortages. For patients in group 1, amphotericin B was usually only available to those patients who could afford to purchase it from private pharmacies. For patients in group 2, the CM-DTP provided amphotericin B for 7–14 days, fluconazole, IV fluids, therapeutic LPs, electrolyte supplementation and laboratory monitoring. The CM-DTP provided supplemental supplies and treatments when the hospital was out of stock.

We evaluated treatments given to patients in groups 1 and 2 to determine how well Ugandan clinical guidelines were followed with respect to CM treatment using combination therapy with amphotericin B plus fluconazole (Table 5). Among patients with presumed plus confirmed CM who were treated with antifungal therapy, 49.6% received combination amphotericin B and fluconazole in group 1 and 74.7% received combination therapy in group 2 (p <0.001). Most patients who were treated for CM, but did not receive combination therapy, were treated with fluconazole monotherapy, which has been shown to have worse outcomes compared to combination therapy [9,18,19]. These results suggest that for patients who received antifungal therapy to treat presumed or confirmed CM, the CM-DTP led to more appropriate use of combination antifungal therapy in accordance with Ugandan clinical guidelines. Patients with confirmed CM in group 1 and group 2 received similar treatments, with most receiving combination antifungal therapy, consistent with Ugandan guidelines [16]. It is likely that patients who were able to pay for diagnostic testing were also able to pay for combination antifungal therapy. In contrast, those who were treated for CM but did not receive diagnostic testing to confirm CM were more likely to receive suboptimal therapy with fluconazole only or sporadic treatment as noted in some patient charts.

**Table 5:** Impact of the CM-DTP on treatment.

		Group 1 2015-2017		Group 2 2017-2019		
CM Treatment		N	%	N	%	P-value
Amphotericin B and Fluconazole		40	83.3	89	89.9	0.38
Confirmed CM Cases		48		99		
Amphotericin B and Fluconazole		63	49.6	109	74.7	**<0.001**
Confirmed plus Presumed CM Cases		127		146		
Monotherapy with Fluconazole		6	12.5	5	5.05	0.18
Confirmed CM Cases		48		99		
Monotherapy with Fluconazole		37	29.1	7	4.79	**<0.001**
Confirmed plus Presumed CM Cases		127		146		
Treatment with Antibiotics		44	91.7	78	78.8	0.06
Confirmed CM Cases		48		99		
Treatment with LP		34	70.8	96	96.9	**<0.001**
Confirmed CM Cases		48		99		
		**N**	Mean*	**N**	Mean*	**P-value**
Number of LPs		41	1.21 (SD 0.48)	251	2.61 (SD 1.14)	**<0.001**
All cases with LPs		34		96		
**Number of Antibiotics**	**Group 1** **N = 44**	**Group 2** **N = 81**	**P-value**	**Group 1 Died**	**Group 2 Died**	**P-value**
≤ 2	16 (36.4%)	68 (84%)	**<0.001**	6 (37.5%)	27 (39.7%)	>0.99
> 2	28 (63.6%)	13 (16.1%)	**<0.001**	17 (60.7%)	7 (53.8%)	>0.99

*Mean number of LPs in cases where LP was performed.

### Improved mortality after CM-DTP implementation

We performed analyses to determine if implementation of the CM-DTP was associated with improved mortality outcomes. Because the diagnosis of CM was suboptimal in group 1, due to the lack of laboratory testing, we performed analyses to determine the effect of the CM-DTP on mortality for 1) those with confirmed plus presumed CM who were treated with antifungal therapy, 2) those with laboratory confirmed CM, and 3) those with all causes of meningitis (Table 6). After implementation of the CM-DTP, mortality decreased for those with confirmed plus presumed CM (all those treated for CM with antifungal therapy) from 52% to 39%, (p = 0.04). Improved diagnosis and access to appropriate treatment may have improved mortality outcomes among those in group 2. Among patients with confirmed CM, there was a 12.9% decrease in mortality from group 1 to group 2, but this difference was not significant. However, all patients with confirmed CM in both groups received similar combination antifungal therapy according to Ugandan guidelines. We found the mortality for all causes of meningitis was lower in group 2, when compared with group 1 (29.9% vs. 42.8%, respectively, p = 0.04). Since the CM-DTP focused only on CM care, it is possible that the decrease in overall meningitis mortality may be attributed to improved diagnosis and treatment of CM because the majority of meningitis cases were presumed or confirmed CM.

**Table 6:** Impact of the CM-DTP on mortality.

Mortality	Group 1 2015-2017		Group 2 2017-2019				
Group	Patients	Deaths N (%)	Patients	Deaths N (%)	OR	CI	P-value
All Meningitis	215	92 (42.8%)	281	84 (29.9%)	**0.57**	**0.39-0.83**	**0.003**
Confirmed & Presumed CM	127	66 (52.0%)	146	57 (39.0%)	**0.59**	**0.36-0.99**	**0.04**
Confirmed CM	48	26 (54.2%)	99	41 (41.4%)	0.60	0.28-1.27	0.16

### Predictors and associations related to CM mortality

We performed analyses of our data to identify risk factors associated with mortality. Among the CM confirmed patients from group 2, we found that a GCS <15 was a significant predictor of death. Those who had a GCS <15 had increased odds of death compared to those who had a normal GCS (OR: 4.28, CI: 1.63–11.9, p = 0.001, Table 7). We also found that an increased number of LPs performed was associated with decreased mortality.

**Table 7:** Factors associated with mortality among patients with confirmed CM in group 2.

Patients with Confirmed CM	N (%)	Death N (%)	OR	Adjusted OR	P-value	CI
GCS <15	45/91 (49.5%)	26/45 (28.6%)	**4.28**		**0.001**	**1.63-11.90**
GCS =15	46/91 (50.5%)	11/46 (23.9%)	1			
Any Antifungal	93/96 (96.9%)	38/93 (40.9%)	0		0.07	0-1.77
No Antifungal	3/96 (3.1%)	3/3 (100%)	1			
Antibiotics > 2	13/81 (16.0%)	7/13 (53.8%)	1.77		0.35	0.54-5.85
				2.46Ψ	0.19Ψ	0.63-9.56Ψ
				3.82**‡**	0.08**‡**	0.86-16.96**‡**
Antibiotics ≤ 2	68/81 (84.0%)	27/68 (39.7%)	1			
Patients with LPs	96/99 (97.0%)	39/96 (40.6%)	0.37		0.58	0.006-7.38
Patients without LPs	3/95 (3.0%)	2/3 (66.6%)	1			
Number of LPs per Patient (Mean 2.24; SD 1.14)			0.64		0.02	0.44-0.92
				**0.62** Ψ	**0.03** Ψ	**0.39- 0.96** Ψ
				0.80**‡**	0.36**‡**	0.49- 1.30**‡**

*Denominators are not equal in each category due to missing/undocumented data.

Ψ OR Adjusted for GCS.

**‡** OR Adjusted for GCS and days of hospitalization.

Among patients with confirmed CM, the average number of LPs performed increased from 1.21 in group 1 to 2.61 in group 2 (p <0.001, Table 5). Fewer patients died who received an LP in group 2 (42.4%), compared to group 1 (58.3%, p = 0.08). Among those in group 2, multivariate logistic regression was conducted, which found the number of LPs was not significantly different between those who lived and died. However, our data showed that those who had more LPs had lower odds of death compared to those who had fewer LPs, but this was not statistically significant after adjusting for confounders of GCS<14 and length of hospitalization. (OR: 0.80, CI: 0.49–1.30, p = 0.36).

The number of different antibiotics ordered for patients with confirmed CM was assessed to determine if this was associated with mortality. The overall number of antibiotics ordered decreased in group 2 compared to group 1 as fewer patients in group 2 had more than 2 antibiotics prescribed compared to group 1 (p<0.001; Table 5). In group 2, those who had more than 2 antibiotics prescribed had an increased odds of death (OR: 3.82, CI:0.86–16.96) after controlling for confounders of GCS <14 and hospitalization days, but this was not statistically significant (p = 0.08).

## Discussion

The CM-DTP was developed to meet the challenges of implementing quality CM care in rural Uganda based on Ugandan guidelines. Our study found that this program, in which care was provided by local clinicians, improved the diagnosis and treatment of CM, and led to improved mortality outcomes among patients with meningitis. These results suggest that high quality treatment of CM in rural settings is feasible.

The CM-DTP led to improved diagnosis of CM, primarily by making the CrAg LFA test widely available at LRRH. The CrAg LFA test is inexpensive ($2–4 USD per test) [20,21] and can be easily performed at the bedside to make a rapid diagnosis of CM. In contrast, the LA test is more expensive and requires samples be sent to an outside laboratory. The LFA test has a 99.3% sensitivity and 99.1% specificity [6,22] while the LA test has a sensitivity of 97–97.8% and specificity of 85.9–100% [23]. Thus, the LFA test is a practical choice for making CM diagnoses in rural settings with its high degree of feasibility and reliability. Providing free LFA testing was a key factor for improving the number of tests conducted and improved the quality of testing. Our results suggest that the use of the rapid and accurate CrAg LFA test led to increased delivery of appropriate treatment based on Ugandan guidelines. Our results also suggest that obtaining a confirmed diagnosis of CM led to decreased use of antibiotics in these patients. This decrease in the use of unnecessary antibiotics in patients with CM not only lowers the chances of creating antibiotic-resistant pathogens but reduces costs for the hospital and patient.

The CM-DTP led to improved mortality outcomes which was even evident among the set of all patients with meningitis when comparing groups 1 and 2. This improved mortality is likely due to the high prevalence of CM among all patients with meningitis and the increased diagnosis and appropriate treatment of CM in group 2. Also, the use of the CrAg LFA helped to exclude CM in patients with other forms of meningitis which may have led to a more focused treatment for other treatable causes, such as bacterial meningitis. The in-hospital mortality for those with confirmed CM in group 2 was 41.4%. This mortality is similar to other CM treatment studies conducted in sub-Saharan Africa which reported short-term mortality ranging from 21.4–55.9% [8,19,24,25]. This study also supports past research which found that altered mental status was associated with increased mortality [26,27]. Our results are also consistent with previous reports that increased number of LPs (diagnostic and therapeutic) was associated with improved mortality [26,28], although our results were not statistically significant.

## Limitations

Our study was limited by the small sample size for patients with confirmed CM in group 1, which limited the analyses to determine differences in risk factors associated with mortality between the groups. Additionally, missing or incomplete hospital records limited our ability to conduct analyses of morbidity between the groups and to confirm whether patients actually received ordered treatments, specifically in group 1. For group 2, there were brief periods, lasting from a few days to a few weeks, when stockouts of antifungal medications or electrolyte replacement therapies occurred. In those instances, all efforts were made to get supplies quickly from alternative sources from within or outside of Uganda. Also, because this study compared historical groups of patients, there may be unrecognized confounders contributing to improved outcomes in group 2, beyond the improved diagnostic testing and treatment that we attribute to the implementation of the CM-DTP.

## Conclusion

Overall, our study demonstrated that high quality and effective treatment of CM by local clinicians in rural areas, is feasible and should serve as a catalyst for implementation of similar programs in other rural regions of sub-Saharan Africa, where efforts to provide the resources and infrastructure for quality CM care should be prioritized.

## Supporting information

S1 Table Primary data for patients with confirmed or presumed CM. The patients listed in this Supplement were diagnosed with confirmed or presumed CM as defined in the Methods. Patients included in this Supplement from cohort 1 were admitted to the hospital from February 2015 to February 2017, except two patients who were admitted in late January 2015. Patients in cohort 2 were admitted from February 2017 to February 2019. The other diagnoses listed are the confirmed or suspected diagnoses during their hospitalization, based on their hospital charts.
(XLSX)

S2 Table Primary data for patients with non-CM meningitis. The patients listed in this Supplement were hospitalized for meningitis, but did not have confirmed or presumed CM. Patients included in this Supplement from cohort 1 were admitted to the hospital with non-CM meningitis from February 2015 to February 2017, except one patient who was admitted in January 2015. Patients with non-CM meningitis in cohort 2 were admitted from February 2017 to February 2019. The diagnoses listed are the confirmed or suspected diagnoses during their hospitalization, based on their hospital charts.
(XLSX)

## References

[1] Joint United Nations Programme On Hiv/Aids (Unaids). 90-90-90 An Ambitious Treatment Target To Help End The Aids Epidemic 2018. Available From: Http://Www.Unaids.Org/Sites/Default/Files/Media_Asset/90-90-90_En.Pdf.

[2] Rajasingham R, Smith Rm, Park Bj, Jarvis Jn, Govender Np, Chiller Tm, Et Al. Global Burden Of Disease Of Hiv-Associated Cryptococcal Meningitis: An Updated Analysis. The Lancet Infectious Diseases. 2017;17(8):873–81. Epub 2017/05/10. doi: 10.1016/S1473-3099(17)30243-8 ; Pubmed Central PMCID: PMC5818156. 28483415 PMC5818156

[3] An Kiragga, Mubiru F Kambugu Ad, Kamya Mr, Castelnuovo B. A Decade Of Antiretroviral Therapy In Uganda: What Are The Emerging Causes Of Death? Bmc Infect Dis. 2019;19(1):77. Epub 2019/01/23. doi: 10.1186/s12879-019-3724-x ; Pubmed Central PMCID: PMC6341568. 30665434 PMC6341568

[4] Center For Disease Control (Cdc). Cryptococcus: Screening For Opportunistic Infection Among People Living With Hiv/ Aids2018. Available From: Https://Www.Cdc.Gov/Fungal/Pdf/At-A-Glance-508c.Pdf.

[5] Beyene T, Woldeamanuel Y, Asrat D, Ayana G, Boulware Dr. Comparison Of Cryptococcal Antigenemia Between Antiretroviral Naive And Antiretroviral Experienced Hiv Positive Patients At Two Hospitals In Ethiopia. Plos One. 2013;8(10):E75585. Epub 2013/10/15. doi: 10.1371/journal.pone.0075585 ; Pubmed Central PMCID: PMC3790840. 24124498 PMC3790840

[6] Rajasingham R, Meya Db, Greene Gs, Jordan A, Nakawuka M, Chiller Tm, Et Al. Evaluation Of A National Cryptococcal Antigen Screening Program For Hiv-Infected Patients In Uganda: A Cost-Effectiveness Modeling Analysis. Plos One. 2019;14(1):E0210105. Epub 2019/01/11. doi: 10.1371/journal.pone.0210105 ; Pubmed Central PMCID: PMC6328136. 30629619 PMC6328136

[7] Meiring S, Fortuin-De Smidt M, Kularatne R, Dawood H, Govender Np. Prevalence And Hospital Management Of Amphotericin B Deoxycholate-Related Toxicities During Treatment Of Hiv-Associated Cryptococcal Meningitis In South Africa. Plos Negl Trop Dis. 2016;10(7):E0004865. Epub 2016/07/29. doi: 10.1371/journal.pntd.0004865 ; Pubmed Central PMCID: PMC4965057. 27467556 PMC4965057

[8] Rkk Patel, Leeme T, Azzo C, Tlhako N, Tsholo K, Tawanana Eo, Et Al. High Mortality In Hiv-Associated Cryptococcal Meningitis Patients Treated With Amphotericin B-Based Therapy Under Routine Care Conditions In Africa. Open Forum Infect Dis. 2018;5(11):Ofy267. doi: 10.1093/ofid/ofy267 Ecollection 2018 Nov. 30488038 PMC6251350

[9] Beyene T, Zewde Ag, Balcha A, Hirpo B, Yitbarik T, Gebissa T, Et Al. Inadequacy Of High-Dose Fluconazole Monotherapy Among Cerebrospinal Fluid Cryptococcal Antigen (Crag)-Positive Human Immunodeficiency Virus-Infected Persons In An Ethiopian Crag Screening Program. Clin Infect Dis. 2017;65(12):2126–9. Epub 2017/10/12. doi: 10.1093/cid/cix613 ; Pubmed Central PMCID: PMC5850618. 29020172 PMC5850618

[10] Loyse A, Burry J, Cohn J, Ford N, Chiller T, Ribeiro I, Et Al. Leave No One Behind: Response To New Evidence And Guidelines For The Management Of Cryptococcal Meningitis In Low-Income And Middle-Income Countries. The Lancet Infectious Diseases. 2018. Epub 2018/10/23. doi: 10.1016/S1473-3099(18)30493-6 . 30344084

[11] Sf Molloy, Chiller T Greene Gs, Burry J Govender Np, Kanyama C Et Al. Cryptococcal Meningitis: A Neglected Ntd? Plos Neglected Tropical Diseases. 2017;11(6):E0005575. doi: 10.1371/journal.pntd.0005575 28662028 PMC5490932

[12] The World Bank Group. World Urbanization Prospects 2018 [Cited 2020 April 22, 2020]. Available From: Https://Data.Worldbank.Org/Indicator/Sp.Urb.Totl.In.Zs?Locations=Zg-Ug.

[13] The World Bank Group. Rural Population–Uganda 2019 [May 7, 2020]. Available From: Https://Data.Worldbank.Org/Indicator/Sp.Rur.Totl.Zs?Locations=Ug.

[14] Nabongo B. Personal Communication. 25 January 2019.

[15] Kc Titeca, Theophile. An Lra For Everyone: How Different Actors Frame The Lord’s Resistance Army. African Affair. 2014;454(114):92–114. doi: 10.1093/Afraf/Adu081

[16] Republic Of Uganda Ministry Of Health. Uganda Clinical Guidelines 2016. National Guidelines For Management Of Common Conditions. 6th Edition Ed. Kampala, Uganda: Ministry Of Health Uganda; 2016. P. 1142.

[17] R Core Team. R: A Language And Environment For Statistical Computing. 3.6.3 Ed. Vienna, Austria: R Foundation For Statisical Computing; 2020.

[18] Jc Nussbaum, Jackson A, Namarika D, Phulusa J, Kenala J, Kanyemba C, Et Al. Combination Flucytosine And High-Dose Fluconazole Compared With Fluconazole Monotherapy For The Treatment Of Cryptococcal Meningitis: A Randomized Trial In Malawi. Clin Infect Dis. 2010;50(3):338–44. Epub 2009/12/30. doi: 10.1086/649861 ; Pubmed Central PMCID: PMC2805957. 20038244 PMC2805957

[19] Sf Molloy, Kanyama C, Heyderman Rs, Loyse A, Kouanfack C, Chanda D, Et Al. Antifungal Combinations For Treatment Of Cryptococcal Meningitis In Africa. The New England Journal Of Medicine. 2018;378(11):1004–17. Epub 2018/03/15. doi: 10.1056/NEJMoa1710922 . 29539274

[20] Chipungu C, Veltman Ja, Jansen P, Chiliko P, Lossa C, Namarika D, Et Al. Feasibility And Acceptability Of Cryptococcal Antigen Screening And Prevalence Of Cryptocococcemia In Patients Attending A Resource-Limited Hiv/Aids Clinic In Malawi. J Int Assoc Provid Aids Care. 2015;14(5):387–90. Epub 2015/07/04. doi: 10.1177/2325957415592475 . 26139095

[21] At Mamuye, Bornstein E, Temesgen O, Blumberg Hm, Kempker Rr. Point-Of-Care Testing For Cryptococcal Disease Among Hospitalized Human Immunodeficiency Virus-Infected Adults In Ethiopia. Am J Trop Med Hyg. 2016;95(4):786–92. Epub 2016/08/17. doi: 10.4269/ajtmh.15-0857 ; Pubmed Central PMCID: PMC5062773. 27527636 PMC5062773

[22] Rajasingham R, Wake Rm, Beyene T, Katende A, Letang E, Boulware Dr. Cryptococcal Meningitis Diagnostics And Screening In The Era Of Point-Of-Care Laboratory Testing. J Clin Microbiol. 2019;57(1). Epub 2018/09/28. doi: 10.1128/JCM.01238-18 ; Pubmed Central PMCID: PMC6322457. 30257903 PMC6322457

[23] Boulware Dr, Ma Rolfes, Rajasingham R, Von Hohenberg M, Qin Z, Taseera K, Et Al. Multisite Validation Of Cryptococcal Antigen Lateral Flow Assay And Quantification By Laser Thermal Contrast. Emerg Infect Dis. 2014;20(1):45–53. Epub 2014/01/01. doi: 10.3201/eid2001.130906 ; Pubmed Central PMCID: PMC3884728. 24378231 PMC3884728

[24] Bo Adeyemi, Ross A. Management Of Cryptococcal Meningitis In A District Hospital In Kwazulu-Natal: A Clinical Audit. African Journal Of Primary Health Care & Family Medicine. 2014;6(1):E1–6. Epub 2014/01/01. doi: 10.4102/Phcfm.V6i1.672 ; Pubmed Central PMCID: PMC4502904. 26245410 PMC4502904

[25] Tk Nyazika, Hagen F, Machiridza T, Kutepa M, Masanganise F, Hendrickx M, Et Al. Cryptococcus Neoformans Population Diversity And Clinical Outcomes Of Hiv-Associated Cryptococcal Meningitis Patients In Zimbabwe. J Med Microbiol. 2016;65(11):1281–8. Epub 2016/09/18. doi: 10.1099/jmm.0.000354 . 27638836

[26] Ma Rolfes, Kh Hullsiek, Rhein J, Nabeta Hw, Taseera K, Schutz C, Et Al. The Effect Of Therapeutic Lumbar Punctures On Acute Mortality From Cryptococcal Meningitis. Clin Infect Dis. 2014;59(11):1607–14. Epub 2014/07/25. doi: 10.1093/cid/ciu596 ; Pubmed Central PMCID: PMC4441057. 25057102 PMC4441057

[27] Je Vidal, Boulware Dr. Lateral Flow Assay For Cryptococcal Antigen: An Important Advance To Improve The Continuum Of Hiv Care And Reduce Cryptococcal Meningitis-Related Mortality. Rev Inst Med Trop Sao Paulo. 2015;57 Suppl 19:38–45. Epub 2015/10/16. doi: 10.1590/S0036-46652015000700008 ; Pubmed Central PMCID: PMC4711197. 26465368 PMC4711197

[28] Meda J, Kalluvya S, Downs Ja, Chofle Aa, Seni J, Kidenya B, Et Al. Cryptococcal Meningitis Management In Tanzania With Strict Schedule Of Serial Lumber Punctures Using Intravenous Tubing Sets: An Operational Research Study. Journal Of Acquired Immune Deficiency Syndromes (1999). 2014;66(2):E31–6. Epub 2014/03/29. doi: 10.1097/QAI.0000000000000147 . 24675586

